# Multimodal digital assessment of depression with actigraphy and app in Hong Kong Chinese

**DOI:** 10.1038/s41398-024-02873-4

**Published:** 2024-03-18

**Authors:** Jie Chen, Ngan Yin Chan, Chun-Tung Li, Joey W. Y. Chan, Yaping Liu, Shirley Xin Li, Steven W. H. Chau, Kwong Sak Leung, Pheng-Ann Heng, Tatia M. C. Lee, Tim M. H. Li, Yun-Kwok Wing

**Affiliations:** 1 Li Chiu Kong Family Sleep Assessment Unit, Department of Psychiatry, Faculty of Medicine, The Chinese University of Hong Kong, Shatin, Hong Kong SAR, China; 2 Department of Psychiatry, Fujian Medical University Affiliated Fuzhou Neuropsychiatric Hospital, Fuzhou, China; 3 Center for Sleep and Circadian Medicine, The Affiliated Brain Hospital of Guangzhou Medical University, Guangzhou, Guangdong China; 4 State Key Laboratory of Brain and Cognitive Sciences, The University of Hong Kong, Hong Kong SAR, China; 5 Sleep Research Clinic and Laboratory, Department of Psychology, The University of Hong Kong, Hong Kong SAR, China; 6 Department of Applied Data Science, Hong Kong Shue Yan University, Hong Kong SAR, China; 7 Department of Computer Science and Engineering, The Chinese University of Hong Kong, Hong Kong SAR, China; 8 Department of Psychology, The University of Hong Kong, Hong Kong SAR, China

**Keywords:** Diagnostic markers, Depression

## Abstract

There is an emerging potential for digital assessment of depression. In this study, Chinese patients with major depressive disorder (MDD) and controls underwent a week of multimodal measurement including actigraphy and app-based measures (D-MOMO) to record rest-activity, facial expression, voice, and mood states. Seven machine-learning models (Random Forest [RF], Logistic regression [LR], Support vector machine [SVM], K-Nearest Neighbors [KNN], Decision tree [DT], Naive Bayes [NB], and Artificial Neural Networks [ANN]) with leave-one-out cross-validation were applied to detect lifetime diagnosis of MDD and non-remission status. Eighty MDD subjects and 76 age- and sex-matched controls completed the actigraphy, while 61 MDD subjects and 47 controls completed the app-based assessment. MDD subjects had lower mobile time (*P* = 0.006), later sleep midpoint (*P* = 0.047) and Acrophase (*P* = 0.024) than controls. For app measurement, MDD subjects had more frequent brow lowering (*P* = 0.023), less lip corner pulling (*P* = 0.007), higher pause variability (*P* = 0.046), more frequent self-reference (*P* = 0.024) and negative emotion words (*P* = 0.002), lower articulation rate (*P* < 0.001) and happiness level (*P* < 0.001) than controls. With the fusion of all digital modalities, the predictive performance (F1-score) of ANN for a lifetime diagnosis of MDD was 0.81 and 0.70 for non-remission status when combined with the HADS-D item score, respectively. Multimodal digital measurement is a feasible diagnostic tool for depression in Chinese. A combination of multimodal measurement and machine-learning approach has enhanced the performance of digital markers in phenotyping and diagnosis of MDD.

## Introduction

Depression is the leading cause of health-related burden globally, affecting an estimated 300 million population [1]. The Hong Kong Mental Morbidity Survey showed that less than 30% of people with common mental disorders had sought help from mental health services [2]. A lack of awareness, stigma [3], and inaccessibility may all contribute to the low rate of help-seeking behaviors. The current gold standard for the diagnosis of depression relies on clinical interview, which serves as a major access block. The insufficient mental health resources are particularly accentuated in a number of Asian and developing regions [4]. In the past decade, there has been a surge of interest in digital phenotyping as a promising, revolutionary, and cost-effective solution [5]. Applying multimodal digital assessment together with artificial intelligence could provide continuous, unobtrusive, and objective assessment without the clinician’s involvement [6].

Digital phenotyping includes a series of digital markers, as depression is more than simply sadness but encompasses a series of physiological, cognitive, mood, and rest-activity changes. For example, emerging data suggested the validity of automatic facial expression analysis for differentiating depression severity [7]. Prosodic language features have been commonly observed in depression [8]. Other passive digital features including sleep, physical activity, location, and phone use data were also studied [9]. The integration of various multimodal digital markers potentially offers a better discriminative power than a single modality [10, 11].

However, there are several gaps regarding the application of digital phenotyping in clinical settings. Few studies have reported the findings of integrating both passive and active features (such as language and facial features) in assessing depression. Besides, the cross-cultural/ethnicity differences in language [12] and lifestyle may suggest the need for local development and validation of digital phenotyping systems. In this study, we utilized a one-week multimodal measurement (actigraphy and our self-developed mobile app named D-MOMO to record rest-activity, facial expressions, voice, and subjective mood state) to assess depression in Hong Kong Chinese.

## Methods

### Study design

The study design is a case–control study that was conducted between June 2021 and March 2023. The study was conducted in compliance with the Declaration of Helsinki, and approved by the Joint Chinese University of Hong Kong—New Territories East Cluster Clinical Research Ethics Committee (Ref No: 2020.492). Informed written consent was obtained from all subjects.

### Study population

The majority of the MDD subjects were recruited from a regional public psychiatric clinic in Hong Kong. These subjects were clinically diagnosed by their attending psychiatrists. In addition, around 22% of the MDD subjects were recruited from the community based on the Structured Clinical Interview for DSM-5—Clinician Version (SCID-5-CV) [13] by a trained medical researcher. The controls were recruited from both the community and sleep centers. MDD patients and controls aged 18 to 65 were recruited. The controls were free from psychiatric diagnosis based on the SCID-5 interview. Exclusion criteria for all study subjects were: (1) Lifetime history of bipolar disorder, schizophrenic-spectrum disorder, dementia, intellectual disability, and neurological disorders; (2) Presence of clear confounding factors (e.g., face injury, speech disorder, night shift workers and motor deficits).

### Multimodal measurement via the D-MOMO app and actigraphy

The Android version of D-MOMO was available in June 2021, and the iOS version was published in April 2022. The app includes a mood diary with a sampling rate of 4 times per day for continuous 7 days. The interval between two time points was 4 h. Subjects preset their first recording time according to their preference and the rest of the three time slots were then determined automatically. For each time, subjects had 1-h buffer to complete the measurement. The subjects first verbally answered two questions (How is your mood right now? What have you done during the last four hours?) with cameras and microphones on. The self-evaluated happiness level was then measured on a Likert scale (0 = very unhappy to 10 = very happy). Rest-activity pattern was measured by Actiwatch Spectrum Plus or Actiwatch Spectrum PRO, Philips Respironics (1 min per epoch) for continuous 7 days. Subjects were instructed to wear the actigraphy on their non-dominant wrist [14].

### Clinical assessment

A 17-item Hamilton depression scale (17-HDS) was used to assess the severity of depression. A cutoff score of 7 or lower and a lifetime history of MDD was indicative of remission [15]. Subjects also completed the Hospital Anxiety and Depression Scale (HADS) [16] for assessing depressive (HADS-D) and anxiety symptoms (HADS-A). The Insomnia severity index (ISI) [17] and reduced Morningness-eveningness questionnaire (rMEQ) [18] measured the severity of insomnia and chronotype preference.

### Actigraphy data processing

Physical activity and sleep estimation were processed by Philips Actiware software 6. A mobile score was defined by 4 or more activity counts per minute. Then, the proportion of mobile time (%mobile) was calculated. Average activity counts per minute (cpm) reflected the quantitative level of physical activity. Sleep parameters included sleep duration, sleep efficiency, sleep midpoint (midpoint between sleep onset and sleep offset), intra-individual variability of sleep midpoint, and intra-individual variability of sleep duration. The circadian rhythm was analyzed using “cosinor” and “nparACT” R packages to perform the cosinor and nonparametric analysis, respectively [19, 20]. More details were provided in Supplementary Methods.

### Speech analysis

Acoustic features (fundamental frequency [F0] mean, F0 variability, articulation rate, pause duration mean, pause variability, and pause rate) were determined via Praat [21] and Tencent Cloud. As background noise would interfere, we performed speech analysis only in those audio recordings under a quiet environment setting. In addition, we excluded the speech segments without pause, as pause was one of our studied outcomes. More details were provided in Supplementary Methods.

### Natural language processing (NLP)

The transcripts generated by Tencent Cloud were manually checked and revised by research personnel who were native Cantonese speakers. For word segmentation, we employed a deep learning-based Chinese word segmentation engine fastHan [22]. After segmentation, words were translated into linguistic and psychologically meaningful categories using the Chinese version of the Language Inquiry and Word Count (LIWC) dictionary [23]. Two categories (self-reference and negative emotion words) were extracted based on former studies [24, 25]. Then, the proportion of words of each category relative to text length (in percentage) was calculated.

### Facial expression analysis

Facial action units (AU) were extracted via OpenFace 2.2.0 software [26]. AUs are all visually discernible facial movements proposed by Paul Ekman [27], and have been widely used for facial expression analysis [7]. We used the presence information (0 or 1) to calculate the proportion of each AU for each video recording. Then, an average value for each AU was calculated based on all the videos of the whole week. Among nine AUs that are related to emotional expression [28], we selected five of them (AU1: Inner brow-raising, AU4: Brow lowering, AU6: Cheek raising, AU12: Lip corner pulling, AU15: Lip corner depressing) for analysis, as they do not occur in speech-affected facial areas [27]. As the study was conducted during the COVID pandemic period, the facial masks might interfere with facial expression, and only videos without masks were studied.

### Statistical analysis

All data acquired was processed and analyzed using SPSS 27.0 and R software. Descriptive data was presented as means ± SD or frequencies (%). Shapiro–Wilk test was applied to assess normality. To investigate intergroup differences of means between two groups, a two-sample *t*-test and Mann–Whitney *U*-test were used for normally and non-normally distributed continuous variables, respectively. To test intergroup differences of means among three groups, the one-way analysis of variance (ANOVA) test, and Kruskal–Wallis test was applied. The Chi-square test was used to compare the intergroup differences for categorical variables. In post hoc analyses, the Bonferroni post hoc test was used for multiple comparisons. Since age was not matched among the three groups in actigraphy measurement, we performed linear regression analysis for actigraphy data (dependent variable) to study its association with remission/non-remission after adjustment for age and sex. A *P* < 0.05 was considered statistical significance, and hypothesis tests were 2 sided.

For predicting lifetime diagnosis of depression and non-remission, we used R package “cutpointr” to determine the optimal cutoff point with a compromise between sensitivity and specificity (balanced data) or sensitivity and PPV (imbalanced data), when features were one-dimensional. Machine-learning (ML) approach was applied in case of multi-dimensional features, at which the performance of seven most commonly used supervised ML methods for disease prediction (Random Forest [RF], Logistic regression [LR], Support vector machine [SVM], K-Nearest Neighbors [KNN], Decision tree [DT], Naive Bayes [NB], and Artificial Neural Networks [ANN]) [29] was compared using leave-one-out cross-validation. Details of extracted features for prediction were provided in Supplementary Table 1. The validity was assessed by F1-score, sensitivity, specificity, positive predictive value (PPV), and negative predictive value (NPV). More details of statistical analysis were provided in Supplementary Methods.

## Results

### Actigraphy data analysis

**Fig. 1:**
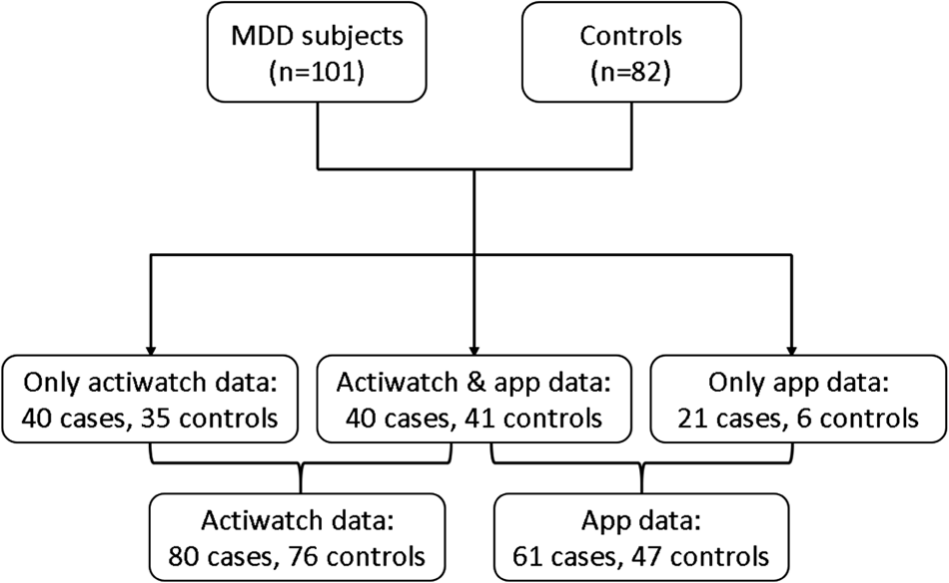
Subject recruitment outline. In total, 80 MDD subjects and 76 controls were included in actigraphy measurement, while 61 MDD subjects and 47 controls completed the app-based measurement.

A total of 80 MDD subjects and 76 controls were included. (Fig. 1) Table 1 summarizes demographic and clinical information. Although the average cpm level during the whole week did not differ between MDD subjects and controls, %mobile was significantly lower in MDD subjects (*P* = 0.006). When we further investigated physical activity levels during active periods and major rest intervals, MDD subjects and controls showed similar average cpm and %mobile levels. The only difference was a significantly longer major rest interval in MDD subjects (*P* = 0.027). %mobile was not associated with psychotropic medication. Age was significantly younger in the non-remitted MDD group than in the remitted group (corrected *P* = 0.020). (Supplementary Table 2) After adjustment for age and sex, both remitted and non-remitted MDD subjects had lower %mobile than controls (remitted MDD: *P* = 0.041; non-remitted MDD: *P* = 0.017).

**Table 1:** Demographic and clinical information, as well as actigraphy data between controls and MDD subjects.

	Controls (*N* = 76)	MDD (*N* = 80)	*P*
Age, mean (SD), y	50.17 (12.04)	49.94 (11.04)	0.76
Female, *N* (%)	48 (63.2)	58 (72.5)	0.21
17-HDS score, mean (SD)	1.61 (2.04)	8.16 (5.75)	<0.001^***^
Remission, %	/	50.0	/
Psychomotor retardation, *N* (%)	/	3 (3.75)	/
Psychomotor agitation, *N* (%)	/	3 (3.75)	/
HADS-D score, mean (SD)	3.36 (2.74)	8.04 (4.07)	<0.001^***^
HADS-A score, mean (SD)	3.70 (2.80)	8.13 (4.24)	<0.001^***^
rMEQ, mean (SD)	16.11 (3.21)	13.22 (4.43)	<0.001^***^
ISI, mean (SD)	5.77 (5.00)	10.45 (5.97)	<0.001^***^
Marital status, %			0.23
Married/Cohabitating	67.1	57.5	
Divorced/Never married/Widowed	32.9	42.5	
Employment status, yes, %	60.3	56.8	0.67
Secondary school or above, %	94.6	94.6	>0.99
Family income monthly <15,000 HKD, %	14.9	33.3	0.010^*^
Medication			/
Antidepressants, %	/	63.7	
Anxiolytics, %	/	22.5	
Antipsychotics, %	/	15.0	
Mood stabilizer, %	/	1.3	
**Physical activity**			
Whole week			
Average cpm, mean (SD)	170.22 (44.40)	159.12 (53.68)	0.13
%mobile, mean (SD)	62.53 (5.45)	59.59 (7.55)	0.006^**^
During active period			
Average cpm, mean (SD)	240.79 (57.15)	239.23 (79.22)	0.64
%mobile, mean (SD)	83.79 (6.86)	83.06 (7.80)	0.85
During rest period			
Average cpm, mean (SD)	20.16 (8.43)	24.65 (15.74)	0.18
%mobile, mean (SD)	17.38 (5.18)	18.08 (8.60)	0.86
Duration, mean (SD), min	457.25 (51.73)	478.57 (58.76)	0.027^*^
**Sleep and circadian markers**			
Sleep estimation			
Sleep duration, mean (SD), minutes	376.84 (45.84)	387.28 (60.41)	0.26
Sleep efficiency, mean (SD), %	82.51 (5.79)	81.03 (8.89)	0.59
Intra-individual variability of sleep duration, mean (SD)	0.14 (0.082)	0.17 (0.10)	0.093
Sleep midpoint, mean (SD), Dec. hours	3.83 (1.03)	4.28 (1.50)	0.047^*^
Intra-individual variability of sleep midpoint, mean (SD), Dec. hours	0.67 (0.55)	0.68 (0.41)	0.57
Circadian rhythm			
Cosinor analysis			
MESOR, mean (SD), counts	170.87 (44.77)	160.26 (53.88)	0.15
Acrophase, mean (SD), Dec. hours	14.92 (1.59)	15.49 (1.62)	0.024^*^
Magnitude, mean (SD), counts	128.97 (47.91)	120.78 (47.08)	0.27
Nonparametric analysis			
IS, mean (SD)	0.56 (0.12)	0.56 (0.13)	0.97
IV, mean (SD)	0.95 (0.24)	0.89 (0.20)	0.12
L5, mean (SD), counts	17.01 (15.03)	19.42 (17.46)	0.65
L5 midpoint, mean (SD), Dec. hours	3.95 (1.72)	4.27 (2.02)	0.14
M10, mean (SD), counts	268.87 (77.50)	252.09 (86.14)	0.20
M10 midpoint, mean (SD), Dec. hours	14.52 (2.20)	15.03 (2.34)	0.14
RA, mean (SD)	0.88 (0.088)	0.86 (0.099)	0.14

*17-HDS* 17-item Hamilton depression scale, *HADS-D* Hospital Anxiety and Depression Scale—Depression subscale, *HADS-A* Hospital Anxiety and Depression Scale—Anxiety subscale, *rMEQ* The reduced Morningness-Eveningness Questionnaire, *ISI* Insomnia Severity Index, cpm activity counts per minute, *MESOR* mean activity count of the fitted 24 h rhythm pattern, *Acrophase* the time of peak activity, *Magnitude* the difference between the peak and MESOR, *IS* interdaily stability, *IV* intradaily variability, *L5* the least active 5-h period, *M10* the most active 10-h period, *RA* relative amplitude. ^***^P < 0.001, ^**^P < 0.01, ^*^P < 0.05.

Later sleep midpoint (*P* = 0.047) and Acrophase (*P* = 0.024) were observed in MDD subjects compared to controls. (Table 1) After adjustment for age and sex, remitted MDD subjects had a delayed Acrophase (*P* = 0.009) and sleep midpoint (*P* = 0.032) than controls, while non-remitted MDD subjects showed a significantly higher intra-individual variability of sleep duration (*P* = 0.003) and marginally higher intra-individual variability of sleep midpoint (*P* = 0.088) compared to controls. Further analysis of sleep onset and sleep offset showed that the delayed sleep midpoint in remitted MDD subjects was due to a later sleep offset (*P* = 0.015) rather than sleep onset (*P* = 0.11). The sleep onset (*P* = 0.31) and sleep offset (*P* = 0.41) were comparable between non-remitted subjects and controls after adjustment for age and sex. Both remitted (ISI: *P* = 0.002; rMEQ: *P* = 0.009) and non-remitted MDD (ISI & rMEQ: *P* < 0.001) were associated with lower rMEQ score (tendency to be more eveningness) and higher ISI score than controls, respectively.

### App measurement

#### Demographic and clinical information, subjective happiness level, and completion rate

Sixty-one MDD subjects and 47 controls completed the app-based measurement. (Fig. 1) Age and sex were matched between two groups (Table 2) or among three groups (Supplementary Table 3). The completion rate of overall 28 data points was 72.89% in MDD subjects and 83.36% in controls. MDD subjects had a lower average subjective happiness level (*P* < 0.001). The average happiness level in remitted (corrected *P* = 0.007) and non-remitted MDD subjects (corrected *P* < 0.001) were both lower than controls. Additionally, the average happiness level was moderately and negatively correlated with 17-HDS score among all subjects (standardized beta coefficients = −0.53, *P* < 0.001).

**Table 2:** Demographic and clinical information, as well as app data between controls and MDD subjects.

	Controls (*N* = 47)	MDD (*N* = 61)	*P*
Age, mean (SD), y	50.21 (11.88)	50.15 (11.28)	0.92
Female, *N* (%)	29 (61.7)	42 (68.9)	0.44
17-HDS score, mean (SD)	1.66 (2.04)	8.66 (6.12)	<0.001^***^
Remission, %	/	51.7	/
Psychomotor retardation, *N* (%)	/	3 (4.9)	/
Psychomotor agitation, *N* (%)	/	2 (3.3)	/
HADS-D score, mean (SD)	3.41 (2.97)	8.24 (4.18)	<0.001^***^
HADS-A score, mean (SD)	4.23 (3.02)	8.44 (4.34)	<0.001^***^
Marital status, %			0.62
Married/Cohabitating	64.4	59.6	
Divorced/Never married/Widowed	35.6	40.4	
Employment status, yes, %	60	53.6	0.52
Secondary school or above, %	91.1	94.7	0.75
Family income monthly <15,000 HKD, %	11.4	32.7	0.012^*^
Medication			/
Antidepressants, %	/	72.1	
Anxiolytics, %	/	26.2	
Antipsychotics, %	/	18.0	
Mood stabilizer, %	/	3.3	
**Subjective happiness level**	6.63 (1.43)	5.02 (1.70)	<0.001^***^
**Facial expression: AU, mean (SD), %**			
*1 (Inner brow raising)*	21.02 (5.30)	23.02 (7.88)	0.14
*4 (Brow lowering)*	21.60 (20.59)	29.70 (20.55)	0.023^*^
*6 (Cheek raising)*	25.52 (26.43)	15.51 (21.01)	0.078
*12 (Lip corner pulling)*	18.21 (17.41)	10.41 (13.21)	0.007^**^
*15 (Lip corner depressing)*	17.46 (8.01)	19.45 (9.14)	0.27
**Acoustic feature**			
F0 mean (Hz), mean (SD)			
Female	169.30 (22.29)	164.12 (24.08)	0.39
Male	110.33 (14.82)	107.01 (13.63)	0.51
F0 variability (Hz), mean (SD)			
Female	51.26 (11.68)	51.19 (10.39)	0.98
Male	36.88 (13.63)	38.77 (12.42)	0.69
Articulation rate, mean (SD)	4.38 (0.51)	3.97 (0.51)	<0.001^***^
Pause duration mean, mean (SD)	0.72 (0.21)	0.82 (0.29)	0.10
Pause variability, mean (SD)	0.31 (0.15)	0.41 (0.27)	0.046^*^
Pause rate, mean (SD)	0.22 (0.085)	0.25 (0.098)	0.13
**NLP feature**			
Negative emotion, mean (SD), %	0.26 (0.45)	0.92 (1.10)	<0.001^***^
Self-reference, mean (SD), %	0.98 (1.11)	1.81 (1.76)	0.018^*^

*17-HDS* 17-item Hamilton depression scale, *HADS-D* Hospital Anxiety and Depression Scale—Depression subscale, *HADS-A* Hospital Anxiety and Depression Scale—Anxiety subscale, *AU* action unit, NLP natural language processing. ^***^P < 0.001, ^**^P < 0.01, ^*^P < 0.05.

#### Facial expression analysis

Fifty-four MDD subjects and 42 controls provided at least one valid video (mean ± SD: 15.5 ± 7.16). MDD subjects showed a significantly higher proportion of AU4 and a lower proportion of AU12 than controls (AU4: *P* = 0.023; AU12: *P* = 0.007). (Table 2) The proportion of AU4 and AU12 was not associated with psychotropic medications. The proportion of AU4 was significantly higher in non-remitted MDD subjects than controls (corrected *P* = 0.004). The proportion of AU4 in remitted MDD subjects was comparable to controls. The proportion of other AUs did not differ among the three groups after multiple testing corrections.

#### Speech analysis

Fifty-four MDD subjects and 41 controls had at least one valid speech segment (mean ± SD: 8.44 ± 5.67). MDD subjects had a significantly lower articulation rate (*P* < 0.001) and higher pause variability (*P* = 0.046) than controls. (Table 2) When the comparison was performed among three groups, the articulation rate was significantly higher in controls compared with both remitted (corrected *P* = 0.042) and non-remitted MDD subjects (corrected *P* < 0.001). In addition, articulation rate seemed to be associated with anxiolytic use (*P* = 0.046). Further analysis by the exclusion of subjects taking anxiolytics, the articulation rate of controls remained significantly higher than both MDD groups (remitted MDD: corrected *P* = 0.029; non-remitted MDD: corrected *P* = 0.036). Pause variability was not associated with psychotropic medications.

#### NLP

The NLP analysis was performed at which MDD subjects had significantly higher percentages of self-reference (*P* = 0.018) and negative emotion words (*P* < 0.001). In addition, remitted MDD subjects showed a higher percentage of self-reference than controls (corrected *P* = 0.048), while non-remitted MDD subjects had a higher percentage of negative emotion words than controls (corrected *P* < 0.001).

#### Multimodal detection of MDD

As shown in Supplementary Table 4, the prediction performance of ANN was generally more favorable compared to other ML methods for both lifetime diagnosis and non-remission with the fusion of all digital modalities. Therefore, ANN was further trained on multi-dimensional features in different modalities. Table 3 lists the performance of predicting lifetime history of MDD among 41 controls and 40 MDD subjects who had both app and actigraphy data. Fusion of all digital modalities resulted in a superior performance (F1-score = 0.81) than that of any single modality including HADS-D. As for predicting non-remission, the multimodal digital features had a F1 performance score of 0.64 and the addition of HADS-D improved the F1-score further to 0.70. (Table 4).

**Table 3:** Performance of predicting lifetime history of MDD.

	No. of subjects (no. of MDD subjects)	No. of features	F1-score	Sensitivity	Specificity	PPV	NPV
Single modality of digital features							
Subjective happiness level^a^	81 (40)	1	0.73	0.83	0.59	0.66	0.77
Actigraphy^b^	74 (36)	17	0.71	0.78	0.61	0.65	0.74
Facial expression^b^	75 (37)	5	0.69	0.86	0.37	0.57	0.74
Voice^b^	69 (33)	4	0.77	0.88	0.64	0.69	0.85
NLP^b^	70 (34)	2	0.75	0.85	0.61	0.67	0.81
All digital modalities^b^	60 (29)	29	0.81	0.86	0.74	0.76	0.85
All digital modalities + HADS-D^b^	56 (26)	30	0.77	0.85	0.70	0.71	0.84
HADS-D^a^	76 (36)	1	0.76	0.75	0.80	0.77	0.78

*NLP* natural language processing, *HADS-D* Hospital Anxiety and Depression Scale—Depression subscale.

^a^Cutpoint optimization for continuous one-dimensional feature.

^b^Artifical Neural Networks.

**Table 4:** Performance of predicting non-remission status of MDD.

	No. of subjects (no. of MDD subjects)	No. of features	F1-score	Sensitivity	Specificity	PPV	NPV
Single modality of digital features							
Subjective happiness level^a^	81 (16)	1	0.49	0.75	0.68	0.36	0.92
Actigraphy^b^	74 (13)	17	0.55	0.92	0.69	0.39	0.98
Facial expression^b^	75 (16)	5	0.41	0.63	0.61	0.30	0.86
Voice^b^	69 (16)	4	0.50	1.00	0.40	0.33	1.00
NLP^b^	70 (16)	2	0.37	1.00	0	0.23	/
All digital modalities^b^	60 (13)	29	0.64	0.69	0.87	0.60	0.91
All digital modalities + HADS-D^b^	56 (11)	30	0.70	0.64	0.96	0.78	0.91
HADS-D^a^	76 (14)	1	0.58	0.64	0.87	0.53	0.92

*NLP* natural language processing, *HADS-D* Hospital Anxiety and Depression Scale—Depression subscale.

^a^Cutpoint optimization for continuous one-dimensional feature.

^b^Artificial neural networks.

## Discussion

In this study, we developed and validated a multimodal digital measurement system (actigraphy and a novel app D-MOMO) to assess depression in Hong Kong Chinese. We found that MDD subjects demonstrated a series of digital features including facial features (more brow lowering and less lip corner pulling), speech features (lower articulation rate, higher pause variability, more self-references and negative emotion words), mood features (lower subjective happiness level), and sleep and circadian features (decreased mobile time, delayed sleep midpoint and Acrophase). For the prediction of a lifetime history of MDD, the performance (F1-score = 0.81) after the fusion of all digital modalities was superior to that of individual modality. On the other hand, the predictive power for non-remission status was relatively lower with the fusion of all digital modalities (F1 score = 0.64) but the predictive power was enhanced by adding HADS-D item (F1-score = 0.70).

Our findings in facial expression analysis supported the mood-facilitation hypothesis [30], at which MDD subjects showed more AU4 (brow lowering) related to negative emotion and less AU12 (lip corner pulling) during positive emotion. Brow lowering is an important component of the omega sign, which was first described by Charles Darwin [31] as a melancholic facial sign [32]. Non-remitted instead of remitted MDD subjects displayed more brow lowering than controls, which suggested that brow lowering was more likely to be a state marker. This was consistent with the hypothesis that brow lowering is activated during hypervigilance or stress response [32, 33].

Similar to another Caucasian study [34], there was a lower articulation rate without a significant increase in pause duration in our MDD subjects. Although only 3 subjects were rated as psychomotor retardation, digital measurement might capture more subtle vocal motor signs. Interestingly, remitted MDD subjects also showed a lower articulation rate. Thus, a lower articulation rate may persist after remission of depression as a trait marker. The findings remained the same after we controlled the use of benzodiazepines [35]. Higher pause variability might reflect more frequent hesitations and stuttering [36]. For physical activity, we found that MDD subjects had decreased mobile time. It was related to longer major rest intervals (i.e., excessive bedtime), which may be contributed by insomnia, anhedonia, or fatigue. The similar cpm level during active period was in contrast with the impression that MDD subjects have reduced motor activity [37]. This might be related to the heterogeneity of depression, as about half of our MDD subjects were in remission.

As for sleep and circadian markers, actigraphy monitoring demonstrated delayed sleep midpoint and Acrophase. This association of eveningness with depression was in line with previous studies [38–40]. Intriguingly, we found a lower rMEQ score but no delay of Acrophase and sleep midpoint in non-remitted MDD subjects, which might be partially explained by their higher intra-individual variability of sleep duration and sleep midpoint. This particular irregular sleep pattern was more marked during a depressive episode [41], and supported by higher ISI scores as well.

More negative emotion words from NLP and lower average happiness levels were found, suggesting a smoldering mood. In line with other Caucasian studies, we found that Chinese MDD subjects tended to use more first-person singular pronouns, which reflected a universal cross-cultural phenomenon of self-focused attention in depressive patients [24, 25, 42].

For predicting the lifetime history of MDD, the best F1-score (0.81) was achieved with the fusion of all digital modalities and ANN machine-learning analysis. The improvement by multimodal fusion was also reported previously [11, 43]. In terms of predicting non-remission, the optimal F1-score (0.70) was reasonably satisfactory, albeit lower than that of lifetime depression. Overall, our results supported the potential of applying digital modalities in detecting both depression state and trait.

The main strength of this study was that our digital system was based on convenient, feasible, and remote measurements without clinician involvement. To our best knowledge, only two studies from Europe [44] and Korea [45] have similar multimodal systems that integrated both passive features (rest-activity pattern) and active features (video or audio). Besides, our app users showed a rather satisfactory adherence rate, which supported the feasibility and application to more subjects. Moreover, the clinical outcome was assessed by trained medical researchers rather than simply based on self-report questionnaires.

There were also some limitations. First, the sample size was only moderate, particularly for multimodal detection. In our future research, we plan to recruit a diverse sample of participants to examine the hypothesis regarding potential differences in the predictive power of multimodal measurements across different ages and genders. Second, age was not matched between remitted and non-remitted MDD subjects in actigraphy data analysis, albeit we made further adjustments in the analysis. Third, speech analysis and facial expression analysis in this study required a state of relative quietness and unmasking that might reduce the convenience of the app measure. Fourth, the potential burden to the subjects (repeated mood diary measurement 4 times per day in the app and 1 week of actigraphy measurement) especially with a vision for future screening and monitoring of depression in a larger population may require further improvement, such as minimization of times and duration of monitoring as well as usage of some smart prompting (e.g., virtual agent like SimSense & MultiSense [46]) in the AI system.

In summary, we have identified a series of digital features of depression. The application of digital modalities with ML provided a good predictive performance for lifetime diagnosis of depression and a relatively lower but still satisfactory performance for predicting non-remission status. Further longitudinal study will be needed to determine whether these digital markers could capture the disease progression and treatment response in depression.

### Supplementary information

supplementary

## Data Availability

The data that support the findings of this study are available from the corresponding author upon reasonable request.
