# Supplementary material for: Multimodal digital assessment of depression with actigraphy and app in Hong Kong Chinese
Source: Transl Psychiatry. 2024 Mar 18;14:150. doi: 10.1038/s41398-024-02873-4 (PMC10948748; doi:10.1038/s41398-024-02873-4)
Supplement: Supplementary file 1 — supplementary [file 41398_2024_2873_MOESM1_ESM.docx]

**Supplementary Methods**

**Actigraphy data processing**

The actigraphy data at the daily level was excluded if the percentage of missing data exceeded 33.3%. Participants who wore the device less than 3 days were excluded. To find active period during a day, both rest interval in the morning and at night should be defined. At least 3 days with defined active period were required for analyzing physical activity during active period. For sleep estimation, a minimum of 3 valid nights were needed. An activity threshold of 40 together with the setting of 10 immobile minutes for sleep onset and sleep offset was used to distinguish sleep from wake status. Then, intra-individual variability of sleep duration was calculated as individual standard deviation of sleep duration divided by individual mean sleep duration.[1] Intra-individual variability of sleep midpoint was calculated as individual standard deviation of sleep midpoint. Cosinor variables included MESOR (mean activity count of the fitted 24-hour rhythm pattern), acrophase (the time of peak activity) and magnitude (the difference between the peak and MESOR). Nonparametric variables included interdaily stability (IS, quantifies the synchronization to the 24h light-dark cycle), intradaily variability (IV, quantifies the rhythm fragmentation), the least active 5-hour period (L5), the most active 10-hour period (M10), and relative amplitude (RA, the relative difference between M10 and L5 in the average 24h pattern).

**Speech analysis**

The pitch analysis was conducted using Praat with pitch-range setting between 50-600 Hz[2-4] and cross-correlation method. Then, the audio data was automatically transcribed into Cantonese using Tencent Cloud. Articulation rate was the number of words over utterance duration (excluding pauses). Silent pauses (pause in spontaneous speech) were identified automatically with 0.25s minimal pause duration[5], then double-checked manually through visual inspection of the spectrogram with the waveform and listening to audio clips on Praat for obvious errors. Pause duration mean was the mean duration of a pause. Pause variability was defined as the dispersion of pause duration (standard deviation). At least two pauses were needed in one speech segment to calculate pause variability. Pause rate was calculated as total length of pauses divided by the total length of speech (including pauses).[6] All values of above acoustic features were averaged over speech segments during one week recording.

**Statistical analysis**

To test potential medication effect (sedating/activating), linear regression model was applied to investigate the association between medications and significant digital features related to psychomotor function (as dependent variable) among MDD subjects after adjustment for 17-HDS score. Linear regression model was also used to correlate the subjective happiness level with 17-HDS score among all subjects.

In terms of machine learning part, we applied pairwise deletion of cases when handling missing data. Feature scaling was used to transform the values of features to a similar scale before certain ML processes (Logistic regression [LR], Support vector machine [SVM], K-Nearest Neighbors [KNN], and Artificial Neural Networks [ANN]). For balanced data (predicting lifetime diagnosis), the best cut-off point was determined using the optimal trade-off between sensitivity and specificity. For imbalanced data (predicting nonremission), the best cut-off point was determined using the optimal trade-off between sensitivity and PPV (i.e., maximum F1-score). Both random oversampling (duplicating samples from the minority class) and undersampling (deleting samples from the majority class) were performed in the training set to handle class imbalance problem. The main R packages involved were “randomForest” for Random Forest (RF), “e1071” for SVM, “caret” for KNN, “party” for Decision tree (DT), “naivebayes” for Naive Bayes (NB), and “neuralnet” for ANN. The code for this study [and training/validation datasets] was available to qualified researchers on reasonable request from the corresponding author.

**Supplementary References**

1. Lemola, S., T. Ledermann, and E.M. Friedman, *Variability of sleep duration is related to subjective sleep quality and subjective well-being: an actigraphy study.* PLoS One, 2013. **8**(8): p. e71292.

2. Postma-Nilsenová, M. and E. Postma, *Auditory perception bias in speech imitation.* Frontiers in Psychology, 2013. **4**.

3. Hussenbocus, A.Y., M. Lech, and N.B. Allen. *Statistical differences in speech acoustics of major depressed and non-depressed adolescents*. in *2015 9th International Conference on Signal Processing and Communication Systems (ICSPCS)*. 2015.

4. He, B., Y. Na, I. Demirkol, and W. Heinzelman. *BaNa: A hybrid approach for noise resilient pitch detection*. in *2012 IEEE Statistical Signal Processing Workshop (SSP)*. 2012.

5. De Jong, N.H., M.P. Steinel, A. Florijn, R.O.B. Schoonen, and J.H. Hulstijn, *Linguistic skills and speaking fluency in a second language.* Applied Psycholinguistics, 2013. **34**(5): p. 893-916.

6. Low, D.M., K.H. Bentley, and S.S. Ghosh, *Automated assessment of psychiatric disorders using speech: A systematic review.* Laryngoscope Investig Otolaryngol, 2020. **5**(1): p. 96-116.

7. Pépiot, E., *Male and female speech: A study of mean f0, f0 range, phonation type and speech rate in parisian French and American English speakers.* Proceedings of the International Conference on Speech Prosody, 2014: p. 305-309.

Supplementary Table 1 Extracted features for multimodal detection of MDD

| Modality | Features |
| --- | --- |
| Subjective happiness level | Subjective happiness level |
| Actigraphy | Average cpm, %mobile, IS, IV, L5, L5 midpoint, M10, M10 midpoint, RA, MESOR, Acrophase, Magnitude, Sleep duration, Sleep efficiency, Intra-individual variability of sleep duration, Sleep midpoint, Intra-individual variability of sleep midpoint |
| Facial expression | AU1 *(Inner brow raising)*, AU4 *(Brow lowering)*, AU6 *(Cheek raising)*, AU12 *(Lip corner pulling)*, AU15 *(Lip corner depressing)* |
| Voice^+^ | Articulation rate, Pause duration mean, Pause variability, Pause rate |
| NLP | Self-reference, Negative emotion |

Cpm: Average activity counts per epoch; MESOR: mean activity count of the fitted 24-hour rhythm pattern; Acrophase: the time of peak activity; Magnitude: the difference between the peak and MESOR; IS: interdaily stability; IV: intradaily variability; L5: the least active 5-hour period; M10: the most active 10-hour period; RA: relative amplitude. NLP: Natural Language Processing

^+^Because there is a marked cross-language sex difference in fundamental frequency [F0][7], F0 mean and F0 variability were not selected in the prediction model.

Supplementary Table 2 Demographic and clinical information among three groups in actigraphy measurement

|  | Controls^1^  (N = 76) | Remitted MDD^2^  (N = 40) | Nonremitted MDD^3^  (N = 40) | Overall P | Post hoc |
| --- | --- | --- | --- | --- | --- |
| Age, mean (SD), y | 50.17 (12.04) | 53.33 (10.54) | 46.55 (10.60) | 0.024 | 2 > 3 |
| Female, N (%) | 48 (63.2) | 26 (65.0) | 32 (80.0) | 0.16 | N.A. |
| 17-HDS score, mean (SD) | 1.61 (2.04) | 3.28 (2.21) | 13.05 (3.61) | < 0.001^***^ | 3 > 2 > 1 |
| Psychomotor retardation, N (%) | / | 1 (2.5) | 2 (5.0) | 1.00 | N.A. |
| Psychomotor agitation, N (%) | / | 0 | 3 (7.5) | 0.24 | N.A. |
| HADS-D score, mean (SD) | 3.36 (2.74) | 6.20 (3.61) | 10.06 (3.59) | < 0.001^***^ | 3 > 2 > 1 |
| HADS-A score, mean (SD) | 3.70 (2.80) | 6.57 (4.27) | 9.79 (3.58) | < 0.001^***^ | 3 > 2 > 1 |
| Marital status, % |  |  |  | 0.49 | N.A. |
| Married/Cohabitating | 67.1 | 57.9 | 57.1 |  |  |
| Divorced/Never married/Widowed | 32.9 | 42.1 | 42.9 |  |  |
| Employment status, yes, % | 60.3 | 52.6 | 61.1 | 0.69 | N.A. |
| Secondary school or above, % | 94.6 | 94.7 | 94.4 | 1.00 | N.A. |
| Family income monthly < 15,000 HKD, % | 14.9 | 32.4 | 34.3 | 0.034^*^ | N.S. |
| Medication |  |  |  |  |  |
| Antidepressants, % | / | 60.0 | 67.5 | 0.49 | N.A. |
| Anxiolytics, % | / | 10.0 | 35.0 | 0.007^**^ | N.A. |
| Antipsychotics, % | / | 7.5 | 22.5 | 0.060 | N.A. |
| Mood stabilizer, % | / | 0 | 2.5 | 0.50 | N.A. |

17-HDS: 17-item Hamilton depression scale; HADS-D: Hospital Anxiety and Depression Scale - Depression subscale; HADS-A: Hospital Anxiety and Depression Scale - Anxiety subscale; ^***^ P < 0.001, ^**^ P < 0.01, ^*^ P < 0.05; N.A.: Not applicable; N.S.: Not significant.

Supplementary Table 3 Demographic and clinical information among three groups in app measurement

|  | Controls^1^  (N = 47) | Remitted MDD^2^  (N = 31) | Nonremitted MDD^3^  (N = 30) | Overall P | Post hoc |
| --- | --- | --- | --- | --- | --- |
| Age, mean (SD), y | 50.21 (11.88) | 53.35 (9.88) | 46.83 (11.83) | 0.13 | N.A. |
| Female, N (%) | 29 (61.7) | 22 (71.0) | 20 (66.7) | 0.70 | N.A. |
| 17-HDS score, mean (SD) | 1.66 (2.04) | 3.55 (2.26) | 13.93 (3.93) | < 0.001^***^ | 3 > 2 > 1 |
| Psychomotor retardation, N (%) | / | 1 (3.2) | 2 (6.7) | 0.95 | N.A. |
| Psychomotor agitation, N (%) | / | 0 | 2 (6.7) | 0.23 | N.A. |
| HADS-D score, mean (SD) | 3.41 (2.97) | 6.32 (3.66) | 10.22 (3.79) | < 0.001^***^ | 3 > 2 > 1 |
| HADS-A score, mean (SD) | 4.23 (3.02) | 6.79 (4.52) | 10.23 (3.37) | < 0.001^***^ | 3 > 2 > 1 |
| Marital status, % |  |  |  | 0.69 | N.A. |
| Married/Cohabitating | 64.4 | 55.2 | 64.3 |  |  |
| Divorced/Never married/Widowed | 35.6 | 44.8 | 35.7 |  |  |
| Employment status, yes, % | 60 | 58.6 | 48.1 | 0.59 | N.A. |
| Secondary school or above, % | 91.1 | 96.6 | 92.9 | 0.79 | N.A. |
| Family income monthly < 15,000 HKD, % | 11.4 | 33.3 | 32.1 | 0.044^*^ | N.S. |
| Medication |  |  |  |  |  |
| Antidepressants, % | / | 67.7 | 76.7 | 0.44 | N.A. |
| Anxiolytics, % | / | 9.7 | 43.3 | 0.003^**^ | N.A. |
| Antipsychotics, % | / | 9.7 | 26.7 | 0.084 | N.A. |
| Mood stabilizer, % | / | 0 | 6.7 | 0.24 | N.A. |

17-HDS: 17-item Hamilton depression scale; HADS-D: Hospital Anxiety and Depression Scale - Depression subscale; HADS-A: Hospital Anxiety and Depression Scale - Anxiety subscale; ^***^ P < 0.001, ^**^ P < 0.01, ^*^ P < 0.05; N.A.: Not applicable; N.S.: Not significant.

Supplementary Table 4 Prediction performance of different ML models after fusion of all digital modalities

| Model | F1-score | Sensitivity | Specificity | PPV | NPV |
| --- | --- | --- | --- | --- | --- |
| *Predicting lifetime history of MDD* |  |  |  |  |  |
| RF | 0.77 | 0.83 | 0.71 | 0.73 | 0.81 |
| LR | 0.69 | 0.72 | 0.65 | 0.66 | 0.71 |
| SVM | 0.76 | 0.72 | 0.84 | 0.81 | 0.76 |
| KNN | 0.76 | 0.76 | 0.77 | 0.76 | 0.77 |
| DT | 0.73 | 0.93 | 0.42 | 0.60 | 0.87 |
| NB | **0.81** | 0.90 | 0.71 | 0.74 | 0.88 |
| ANN | **0.81** | 0.86 | 0.74 | 0.76 | 0.85 |
| *Predicting nonremission* |  |  |  |  |  |
| RF | 0.45 | 1.00 | 0.32 | 0.29 | 1.00 |
| LR | 0.36 | 1.00 | 0 | 0.22 | / |
| SVM | 0.36 | 1.00 | 0 | 0.22 | / |
| KNN | 0.58 | 0.69 | 0.81 | 0.50 | 0.90 |
| DT | 0.51 | 0.77 | 0.66 | 0.38 | 0.91 |
| NB | 0.53 | 0.77 | 0.68 | 0.40 | 0.91 |
| ANN | **0.64** | 0.69 | 0.87 | 0.60 | 0.91 |

Random Forest: RF, Logistic regression: LR, Support vector machine: SVM, K-Nearest Neighbors: KNN, Decision tree: DT, Naive Bayes: NB, Artificial Neural Networks: ANN.
